# ‘I am doing fine only because I have not told anyone’: the necessity of concealment in the lives of people living with HIV in India

**DOI:** 10.1080/13691058.2015.1009947

**Published:** 2015-02-23

**Authors:** Mathew Sunil George, Helen Lambert

**Affiliations:** ^a^ Indian Institute of Public Health, Delhi, India; ^b^ School of Social and Community Medicine, University of Bristol, Bristol, UK

**Keywords:** concealment, stigma, HIV, treatment adherence, India

## Abstract

In HIV prevention and care programmes, disclosure of status by HIV-positive individuals is generally encouraged to contain the infection and provide adequate support to the person concerned. Lack of disclosure is generally framed as a barrier to preventive behaviours and accessing support. The assumption that disclosure is beneficial is also reflected in studies that aim to identify determinants of disclosure and recommend individual-level measures to promote disclosure. However, in contexts where HIV infection is stigmatised and there is fear of rejection and discrimination among those living with HIV, concealment of status becomes a way to try and regain as much as possible the life that was disrupted by the discovery of HIV infection. In this study of HIV-positive women and children in India, concealment was considered essential by individuals and families of those living with HIV to re-establish and maintain their normal lives in an environment where stigma and discrimination were prevalent. This paper describes why women and care givers of children felt the need to conceal HIV status, the various ways in which people tried to do so and the implications for treatment of people living with HIV. We found that while women were generally willing to disclose their status to their husband or partner, they were very keen to conceal their status from all others, including family members. Parents and carers with an HIV-positive child were not willing to disclose this status to the child or to others. Understanding the different rationales for concealment would help policy makers and programme managers to develop more appropriate care management strategies and train care providers to assist clients in accessing care and support without disrupting their lives.

## Abstract (es)

Durante la ejecución de los programas de prevención y cuidado del vih, se alienta a los seropositivos a compartir con otros su estado de salud, con el fin de reducir el contagio y de brindar el apoyo adecuado al paciente. En general, se considera que no revelar tal estado de salud constituye un impedimento para ofrecer medidas preventivas y posibilitar la obtención de cuidados. Asimismo, el supuesto de que la revelación constituye un acto positivo se ve reflejado en aquellas investigaciones que intentan identificar las razones para realizarla y recomendar medidas susceptibles de ser aplicadas a nivel del paciente para promover dicho ejercicio. Sin embargo, en los contextos en que ser seropositivo conlleva estigmatización, temor al rechazo y discriminación, el ocultamiento del estado de salud se vuelve una opción en el sentido de recobrar, en la medida de lo posible, la vida, perturbada a partir del momento en que el paciente se entera de que es seropositivo. La presente investigación estudió a mujeres y a niños seropositivos de India, encontrando que las personas afectadas por la enfermedad y sus familias consideran esencial el ocultamiento para restablecer y preservar la normalidad de sus vidas en un ambiente colmado por el estigma y la discriminación. Este artículo examina las razones por las cuales las mujeres seropositivas y los cuidadores de niños seropositivos se sienten obligados a encubrir su condición, las distintas maneras en que llevan a cabo tal encubrimiento y las implicaciones que esto conlleva en el tratamiento de las personas seropositivas. Los autores encontraron que, si bien las mujeres generalmente están dispuestas a revelar su estado a sus esposos o parejas, tienen mucho interés en ocultarlo a todas las demás personas, incluso a los propios integrantes de su familia. Mientras que, los padres y los cuidadores de niños seropositivos prefieren ocultar dicho estado al niño mismo y al resto de las personas. Una mejor comprensión de las distintas racionalidades vinculadas al ocultamiento de la condición de seropositivo contribuirá a que los formuladores de políticas y los administradores de programas puedan plantear estrategias adecuadas para el cuidado de los seropositivos y para la capacitación de los prestadores de cuidado a fin de que, a su vez, ayuden a sus clientes a acceder al cuidado y a los apoyos disponibles, sin que ello altere sus vidas.

## Abstract (fr)

Généralement, les programmes de prévention et de soin du VIH encouragent les personnes infectées à dévoiler leur statut sérologique dans un objectif de limitation de la propagation du VIH et d'offre de soutien à ces personnes. Le secret de la séropositivité est généralement considéré comme un obstacle aux comportements préventifs et au soutien. La théorie selon laquelle le dévoilement du statut sérologique est bénéfique se reflète également dans les études visant à identifier les déterminants du dévoilement et recommandant des mesures au plan individuel pour favoriser le dévoilement. Cependant, dans les contextes où la stigmatisation et les discriminations vis-à-vis du VIH sont présentes et redoutées par les personnes vivant avec le VIH, la dissimulation de la séropositivité devient un moyen d'expérimenter et de retrouver, autant que possible, la vie qui a précédé le diagnostic. Dans cette étude sur des femmes et des enfants infectés par le VIH en Inde, cette dissimulation est considérée par ces personnes comme essentielle au rétablissement et au maintien d'une vie normale dans un environnement où stigmatisation et discriminations sont omniprésentes. Cet article explique pourquoi les femmes et les tuteurs d'enfants ressentent le besoin de dissimuler la séropositivité, les différentes stratégies pour y parvenir et leur impact sur l'observance des traitements. Notre étude révèle que si les femmes souhaitent, d'une manière générale, révéler leur statut à leur mari ou à leur partenaire, elles tiennent absolument à ce que « les autres », y compris d'autres membres de la famille, continuent de l'ignorer. Les parents/tuteurs d'enfants séropositifs ne souhaitent pas annoncer ce statut à ces enfants. La compréhension des différentes justifications de la dissimulation de la séropositivité devrait permettre l'élaboration de stratégies de prise en charge plus appropriées et la formation des prestataires de soins à l'aide à l'accès aux soins et au soutien pour les patients.

## Introduction

Disclosure of positive HIV status is generally presented as an important step in providing adequate care and support and in prevention of transmission of HIV to sexual partners of those living with HIV (Cole et al. 1996; Hays et al. 1993; UNAIDS 2000). Programmes that provide counselling and testing, as well as care and treatment programmes, tend to emphasise the importance of disclosure of HIV status. Arising from this understanding of disclosure as a positive and necessary step in HIV prevention, care and treatment, studies have been carried out to identify disclosure levels and predictors of disclosure and to suggest ways and means of improving disclosure of status among HIV-positive individuals (Chandwani et al. 2012; Reynolds et al. 2004; Tiyou et al. 2010; Uuskula et al. 2012). Much of this work tends to focus on the HIV-positive person and their behaviours, primarily from an individual perspective, without adequately accounting for the fact that each individual exists and operates within a social network that is relevant for them as well as within a wider sociocultural milieu.

In this paper, we move away from conceptualising disclosure as a behaviour that can be modified by interventions focused on the individual alone and emphasise instead the importance of taking into account the social context of being HIV-positive and its implications when developing interventions that seek to promote the health and wellbeing of people living with HIV.

## Stigma, culpability and the need to conceal

Stigma surrounding various illnesses and the resultant need for concealment and controlling health-related information have been well documented in the sociological literature, with a particular focus on stigma surrounding mental illness (Schneider and Conrad 1980), chronic conditions such as cancer (Baider 2010) and those with a sexual route, including HIV. In his seminal work on the subject, Goffman (1963) defines stigma as an attribute that is deeply discrediting and reduces the bearer of the stigma from a whole and usual person to a tainted, discounted one. Goffman also describes how those whose stigma is yet to be revealed focus on the management of information that ensures that their identity as discreditable is concealed from others.

With the rise of chronic disease and the growth of non-communicable disease epidemiology, emphasis on changing behaviours that carry health risks and on health as a matter of individual responsibility has become increasingly prominent in public health discourse, as reflected in health promotion programmes and preventive services (Ory, Jordan, and Bazzarre 2002). However, implied culpability for failure to alter forms of behaviour that may lead to ill health has only increased the potential for stigmatisation among those with conditions that can be interpreted as related to behavioural ‘choice’. With the absence of effective treatment options in the initial years of the HIV epidemic, the primary focus was on controlling or preventing behaviours that were considered risky (Carey 1999). The perception of HIV-positive individuals as culpable for their own disease status by adopting behaviours that were known to carry risks for infection is exacerbated by the fact that these include forms of behaviour that are widely considered immoral (and sometimes illegal). This tendency to judgment is gendered and is particularly strong in the case of women.

## Sexuality and the stereotyping of HIV infection among women

Women's sexuality has been framed as normatively virtuous, chaste and submissive in many societies. Women who deviate from these societal norms are commonly stigmatised, stereotyped and subjected to pejorative labels (Nelson 2005). Female sexual purity and chastity has been venerated in many cultures, and in Europe in the nineteenth century, sexual desire was posited as something alien to respectable women (Caplan 1987). In India, the organisation of gender hierarchy has been linked to cultural definitions of women's sexuality as all-devouring and indiscriminate (Lambert 2001). The ancient Indian text of *Manusmriti*, for example, portrays women as seducers who are inherently lustful and whom the wise should avoid (Unknown 1886). The associated requirement for women to marry and remain under male control within patriarchal family structures can be seen as an institutionalised solution to this ‘problem’ of female sexuality (Ramasubban 1992). Hindu cultural assumptions further posit women as the repositories of caste integrity, with sex to be enjoyed only within the confines of marriage and as an act to beget offspring for the patriline. Despite wide regional and community variations in specific kinship and marriage practices across India, family honour is generally held to reside in the virtue of its women.

Historically, there has been a constant connection between illness and sinfulness, impurity and guilt across various cultures (Gilman 1988). In particular, sexually transmitted infections (STIs) have carried with them a strong affiliation with deviant and immoral behaviour (Brandt 1987; East et al. 2012). Women with STIs have been considered to deviate from the traditional ideal of being chaste and have been viewed as tainted (Amaro, Raj, and Reed 2001), whereas men who contract STIs are not commonly subjected to humiliating labels and the infection is considered an unwelcome but ‘normal’ outcome of a man's virility (Bassett and Mhloyi 1991; Bolan, Ehrhardt, and Wasserheit 1999). Such double standards with regard to women are not new and have been documented cross-culturally, but they have particular resonance in India due to the specific alignment of moral and family integrity with female sexual chastity.

Having sexual transmission as one of its key modes of transmission, HIV has attracted the additional stigma associated with other STIs, including indiscriminate promiscuity, pollution and uncleanliness (Lawless, Kippax, and Crawford 1996). Thus, women living with HIV are sometimes considered to have failed to uphold traditional values prescribed for women and are hence viewed as immoral and deserving of stigmatisation. The effects of such cultural constructions of women and sexuality have also been seen in the way women who engage in sex work have been regarded as a source of HIV infection by policy makers, especially in the initial days of the epidemic (Patton 1994). While it has been shown that most women in India have contracted HIV from their spouse, this has in no way diminished judgments regarding their sexual morality, with high levels of discrimination being reported, including by family members (Malave et al. 2014; Padyana et al. 2013).

## Stigma and the promotion of disclosure of HIV status

Stigma associated with HIV and AIDS has always been referred to as a serious problem in providing an effective response to the epidemic (Parker and Aggleton 2003). In addition to adversely affecting individual lives, it also acts as an obstacle to providing adequate prevention and care and support services. Several studies in other settings have captured stigma as a major barrier to the provision of adequate care and treatment services to individuals who need them (Coetzee, Kagee, and Vermeulen 2011; Rintamaki et al. 2006; Sankar et al. 2002; Van Tam et al. 2011; Weiser et al. 2003). Stigma towards HIV and AIDS is often conflated with stigma towards sex workers, men who have sex with men, injecting drug use and sex outside what are considered moral and legal limits defined by society (Mahendra et al. 2007). Such multi-layered stigma serves to justify and deepen the stigma among those infected as well as those affected by HIV, including family members and care givers of people living with HIV (Bharat, Aggleton, and Tyrer 2001; Parker and Aggleton 2003; Sontag 1990). It has also been shown that stigma disrupts the ability to form the social networks that may be facilitative of treatment adherence (Coetzee, Kagee, and Vermeulen 2011). Studies have also shown that when people are faced with the choice of either taking their medication and risking discovery as HIV-positive or preserving their social relationships, they often choose to forgo their medication in favour of preserving social capital (Ware, Wyatt, and Tugenberg 2006).

The act of speaking up publicly regarding HIV infection has long been promoted as a key strategy in overcoming shame and addressing stigma and discrimination. HIV-positive persons were encouraged to speak publicly about their experience of living with HIV, stigma and their strategies to normalise their lives in order to benefit others. This approach has also been incorporated into national and international programmes and interventions. The important role of building awareness about HIV and the strategy of speaking publicly about the experience of living with HIV was given a particular focus at the 13^th^ International AIDS conference held at Durban in the year 2000, which had for its theme ‘Breaking the Silence’. Some studies have found that contact with people living with HIV promotes a more tolerant attitude towards them and that the act of speaking out was beneficial and helped to provide psychological release for those who ‘came out’ (Paxton 2002). However, in most cases, HIV-positive speakers were primarily senior activists whose engagement in the public HIV discourse was overtly political, unlike participants in this study who considered the infection as an unwelcome disruption of their ‘normal lives’ and therefore something they were not comfortable with and tried to skirt. Participants in our study also pointed out that accessing and adhering to antiretroviral therapy (ART) was a way to regain the life that had been disrupted by HIV infection. Hence, in a way, disclosure may be counterproductive to the whole point of being on treatment.

## Methods

The data presented in this paper were gathered during fieldwork conducted between January and October 2012 for a study exploring adherence to ART among clients registered at two government-run ART clinics in an urban centre in the state of Tamilnadu, south India. In-depth interviews were conducted with 32 women and 29 children who were within three months of initiating ART. Within these two broad groups, participants were purposively selected from the various sub-categories (Table 1) in order to gain adequate representation from each of them. In addition, 15 key informants with significant local experience in the field of HIV care were interviewed. The participants were not limited to any particular socioeconomic group, but were from across the socioeconomic spectrum of society and came from different parts of the city and in some cases from the neighbouring districts as well.

**Table 1:** Sampling framework.

Women	Husband/partner alive	Widow	Female sex worker	Long-term adherer	Total
	13	10	9	0	32
Children	Parents alive	Foster care (no parents)	Care home-based	Long-term adherer	
	15	8	3	3	29
Others	Healthcare provider	Expert^*^	Health system official	Women living with HIV/AIDS leader	
	8	4	2	1	15

Note: ‘Expert’ refers to key informants who had several years of experience working with women or children living with HIV in the state of Tamilnadu.

Piloting of the interview topic guides was done among a group of four women and two children on ART and refinements were made to the topic guides following participant feedback and review of data. The first author, who had prior experience in conducting interviews with vulnerable groups and was fluent in the participants' native language (Tamil), conducted most of the interviews, with assistance from an experienced field worker for some interviews in the first round of data collection. Three in-depth interviews were conducted at intervals of four months with each of the participants in this study, yielding a total of 146 interviews. This longitudinal method of data collection was designed to capture changes over time with regard to medicine-taking among the respondents. Repeated encounters over time facilitated the building up of rapport and trust with each interviewee, especially children. Interviews were conducted in separate rooms in the clinic, away from other clients and staff, to ensure that respondents were able to speak freely. In most cases, caregivers of children were present and answered on behalf of the child. During the third round of data collection, some of the caregivers agreed to let children talk to the researcher privately. This enabled capturing their perspectives directly, without the presence of a mediator.

All interviews were audio-recorded, transcribed, translated into English and cross-checked against the original recordings. The translated transcripts were coded using the software package Atlas ti 6.2^®^, utilising a reflexive and inductive approach to allow codes and categories to emerge from within the data. The second author independently coded a sub-sample of the transcripts and the categories were compared. Member checking to improve the accuracy and completeness of the findings was carried out by presenting key findings to a sample of all stakeholders at the end of the study and obtaining their feedback, thereby improving the validity of the results (Barbour 2001; Coffey and Atkinson 1996; Lincon and Guba 1985).

Signed consent was obtained from all participants prior to data collection, with parents or primary carers providing consent on behalf of children under 18 years old. The study received ethical approval from the ethics committee of the Public Health Foundation of India. Regulatory permission was obtained from the Tamilnadu State AIDS Control Society, the government agency overseeing all HIV interventions in Tamilnadu.

## Concealment as a key narrative in the effort to regain a normal life

Concealment of HIV status was an overarching concern among the participants from the point of discovery of HIV status up to maintaining oneself on treatment. Earlier studies have shown that the source of HIV infections among married women in India is frequently traceable to their spouses (Gangakhedkar et al. 1997; Ghosh et al. 2011; Newmann et al. 2000; Rogers et al. 2005). With the exception of one participant, all the women in the study who were not sex workers had come to know of their HIV infection after their partner had developed serious AIDS-related complications or had died. Among sex workers who participated in the study, three mentioned that they had discussed their status with their husbands/stable partners since they felt they should know about their infection, while the others had come to know about their infection only when their husbands were very ill from AIDS-related complications. The only participant who discovered her HIV status by herself mentioned that she made an effort to get in touch with her husband who had separated from her to let him know about her status so that he could take the necessary care for himself and his new partner. In general, participants revealed that they disclosed their status to those they felt would be directly affected by their HIV-positive status or ill health, such as their sexual partners or children. Some also revealed that they had confided with people close to them who would not ostracise them (generally a family member or a friend) and would also be able to support them during episodes of illness or visits to the hospital, including by helping them legitimise the alternative explanations they offered and thereby ensuring that their status remained hidden from others.

In spite of this, every participant was extremely concerned about concealment and described various means they employed in order to achieve this. These included hiding their ART registers, decanting their medication and leaving the pill boxes at the clinic, using a generic container to store the pills and coming up with alternate explanations for visits to the clinic and taking medication:

I keep the card in a cover and then keep it inside a cupboard below the sarees. Once a month when I come here, I take it from the cupboard and bring it here.

(Widow living with HIV, W1)

I bring one box from my home, transfer the tablets to it and leave this box here only.

(Widow living with HIV, W6)

There used to be an LIC (insurance) office just opposite this hospital so whenever I come here, I tell people who ask me where I am going that I am going to pay the premium. Luckily no one knows that the office has been shifted from here.

(Caregiver of HIV-positive child, PO9)

They ask me why I am taking these tablets. I tell them that I am taking this for fever.

(Woman living with HIV, WH5)

In situations where the inadvertent disclosure of HIV status was feared either due to being seen with pills or during the act of taking the medication, most of the participants choose not to take their medication rather than risk having to face uncomfortable situations if discovered. In the opinion of some of the participants, advertisements and public health messages about HIV being treatable mention ART and hence being discovered with ART had a clear association with being HIV-positive.

The need for concealment caused people to refuse any free services that had a reference to HIV or AIDS. In our study, most participants choose to pay for travel, in spite of facing financial constraints, rather than accept the free pass (travel document) that was provided for them by the government, since the word AIDS was present in it:

It is printed as AIDS in the pass. So I haven't taken it.

(Woman living with HIV, S4)

I know a lot of people in the train. They might come to know if they see that I have this right. So I am afraid to take it.

(Woman living with HIV, WH4)

However, among a section of the participants there was a fear that in spite of their best efforts they would be discovered and it would lead to unpleasant consequences. This fear made them take extreme care in ensuring that they were meticulous in concealing their status:

Till I get to the hospital the fear will be there. When I come inside and before I enter this building, I will just turn back once to see if anybody is noticing me and then enter quickly.

(Woman living with HIV, WH1)

One participant described how they came close to being discovered by neighbours due to their presence in the hospital. However since it was a large tertiary care institution she was able to explain her presence with other reasons:

Once when I came here one of my neighbours saw me at the entrance. She asked me what I am doing here. I told her I came to see a friend of mine who was admitted in the ward. After that I went around the hospital and came back here only after some time.

(Woman living with HIV, S4)

Some of the participants also spoke of how they felt HIV infection had caused them to do things they would otherwise not do in order to conceal their status and carry on with their normal routine lives:

In a sense this is just cheating right … so many lies. Every time I have to come here I have to say one lie or another. That has become our life now. But what to do.

(Caregiver of HIV-positive child, PO 9)

## Reasons for concealment

Among the various reasons that participants mentioned for concealing their HIV status, the fear of rejection and of being identified as morally lax due to the association of HIV with sexual permissiveness was the most common. Most participants and their families felt that should their status became publicly known about they would be rejected by their friends and other relatives. In order to prevent such an eventuality, participants choose to conceal their HIV status rather than face rejection from those in their immediate social network. All the participants, whether sex workers, widows or women whose partners were alive, reported experiencing felt stigma. Another persistent theme that emerged while discussing the need to conceal was the fear of being identified as promiscuous and having got the disease as a result of some ‘mistake’ on their part:

Nobody knows. I don't like to disclose. If I tell, they will ask me why I have made a mistake.

(Widow living with HIV, W2)

I didn't commit any mistake. God knows that. Somehow I have been given this disease.

(Widow living with HIV, W3)

But why should I tell anyone? It's a shameful thing to say. I don't know what some people will think. I used to feel that if my relatives come to know then they might say this and that. They might talk all sorts of things about me so I feel it's better not to tell this to anyone. Why say all of this.

(Women living with HIV, S4)

The local term for ‘mistake’ or error (*thappu*) has a moral connotation (in this case primarily with implicit reference to sexual conduct). There is a shared assumption that an individual who has acquired HIV infection will be assumed to have committed a socially deviant act (here, marital infidelity) and is thus in some way morally culpable. Most informants feared that being HIV-positive would constitute sufficient ‘evidence’ for others to stigmatise them on these grounds, regardless of whether they had in fact committed such a ‘mistake’. One participant, whose husband had died some years before she discovered her status, described how family, relatives and others in her social network questioned how she could have got such an infection without ‘committing any mistake’ since she was a widow:

They asked me how I got it without doing any wrong since it has been a long time since he passed away.

(Widow living with HIV, W3)

Another participant mentioned how it would have been better to have another disease due to the stigma and shame associated with HIV:

Sometimes I think if it was cancer it would have been better. At least people will show some compassion there. But with HIV you have to hear shameful things being said.

(Woman living with HIV, WH3)

A further reason for having to conceal their HIV status, according to participants, was fear of discrimination. Even those who had not experienced any concrete adverse consequences described how they had heard of HIV-positive individuals being discriminated against and did not want to risk being ostracised by disclosing their status:

I am really scared. I give my phone number here [in the clinic] I am worried that someone will call. If they find out nobody will give me a house, if there is anything nobody will come to our house.

(Woman living with HIV, WH6)

In spite of their efforts to conceal their status, some of the participants spoke about how they had to face stigma and discrimination when their status as HIV-positive individuals was revealed to others inadvertently. One participant spoke of how she and her husband were identified as HIV-positive at the railway station on their way to collect their monthly dose of ART. The railway staff ridiculed both of them and as a result of this they felt a great sense of fear and shame while travelling on the train subsequently. Hence they choose to pay for their tickets from their own funds rather than make use of the railway pass that entitled them to discounted travel tickets:

The staff at the ticket counter looked at our pass and told the others there. Hey look at this, both husband and wife have gone together and got AIDS and he showed our pass to others who were there. We felt so bad that we wanted to commit suicide. After that we never use the pass when we come here. We pay for our journey since we are afraid that if someone will find out about us again, they will make fun of us.

(Woman living with HIV, WH4)

Another participant described how on discovering from her relatives that her mother was HIV-positive, her own daughter refused to speak to her and continues to avoid her as she considers her to be responsible for the shame and boycott that the family faces among their relatives:

Till today she does not talk to me. That is the greatest source of pain for me. My own daughter avoids me. She thinks I am responsible for this situation.

(Widow living with HIV, W 7)

Those who had experienced ostracism as a result of their HIV-positive status tried to limit the damage caused by avoiding as far as possible social networks and locations where their status was known, while preferring to conceal their status in any new social networks.

Sex workers who participated in this study had to further ensure that their status was not publicised in their area of work, as it would lead to losing their clients. This was particularly difficult since other sex workers and in some instances even a few clients had far greater knowledge about HIV infection and ART than the general population. Clients, they felt, were wary of finding any medication with a sex worker (even if it was not ART) since it indicated that something was wrong with her and their first suspicion was that the person was HIV-positive. While women who were not sex workers also had to manage information about their disease status, their immediate social networks of family, friends, colleagues and so on were less exposed to information related to HIV and therefore less aware of the details of HIV infection, medication and related issues. This meant that a sex worker had to take more precautions to ensure that their status as HIV-positive remained unknown to others.

Among healthcare providers, there was a general tendency to label men as guilty and women and children as innocent victims. The fact that most women in India had contracted HIV from their spouse was used to drive home the fact that women were in general innocent of any ‘wrong doing’ and despite being faithful, had contracted the infection from their husbands, who had got it by indulging in some illegal or immoral activity. Sex workers did not feel comfortable to identify themselves as such with the clinic staff precisely because so doing would rob them of the benefit of being labelled as an innocent victim and they would be marked out as among those who were guilty of immoral behaviour, which had led to their being HIV-positive.

## Children and concealment

Disclosure to children who became HIV-positive through transmission from mother to child necessarily entails disclosing their source of infection as well as their own status as HIV-positive and its consequences for care and treatment. With the exception of a single young person, no other child who participated in this study had been told about their status as HIV-positive. In the case of the individual who knew about her infection, the potential route of infection was strongly suspected to be blood transfusion (other members of her family were not HIV-positive and she had received blood from a private hospital some years previously). The other families and caregivers of HIV-positive children concealed the child's status both from the child and from others. Most parents were uncomfortable to talk about their own HIV infection and to answer potential questions that the child might ask. Therefore, parents concealed both their own HIV-positive status and that of their child. Parents and caregivers were highly guarded about the status of their children and did not even disclose it to other relatives. In some cases, the siblings of HIV-positive children who were not HIV-positive were not told what was wrong with their brother or sister. A generic explanation was usually given about the need to visit the hospital regularly and take medication. The primary rationale that was offered by parents and care givers about not disclosing status to the child was that it was too early and the child might not understand what it really meant to be HIV-positive. However, on probing the issue further in subsequent interviews it became apparent that more than the ability of the child to understand, parents and care givers felt uncomfortable to address the issues surrounding the transmission of HIV with their children. Parents in particular felt guilty that they had been the cause of a potentially terminal condition for their own child and found it extremely difficult to face themselves on this count:

Sometimes I feel very sad when I think I myself gave this deadly infection to my own child. This should not happen even to my worst enemies.

(Father of child with HIV, PF1)

While parents and care givers of children preferred to conceal their status children, especially those who were in their teens, had begun to seek answers to why they had to consume medication on a daily basis. This is well illustrated by the case of an orphan who was a long-term adherer who kept asking his uncle, who was the primary care giver, the exact reason he had to take the medicines:

I have been asking my uncle for a long time why I need to take these medicines but he has not really told me.

(Adolescent on ART, PLTO1)

Young people in particular were keen to understand the implications of taking their medication and what really was going on in their lives. However, neither parents, other care givers nor health system representatives were sure about the best way to engage with children on these issues. A recent study in India among HIV-positive adolescents has shown that while parents and caregivers prefer to remain silent on the issue, a majority of the children came to know about their disease status as a result of their inquisitiveness or suspicion during their periodic visits to the clinic, pointing to the need to address this issue in an appropriate manner (Mothi et al. 2012).

## Discussion: reconceiving disclosure and concealment

In this study we have shown how concealment of status is considered essential by HIV-positive individuals who were in receipt of ART in order to maintain their normal lives within pre-existing social networks, given the highly stigmatised nature of HIV infection and the prevalent association in this sociocultural context between being HIV-positive and behaviour that is perceived as immoral. These informants conceived of ART as a means of ensuring that HIV infection did not disrupt their lives further and as enabling them to retain the social relations that were endangered as a result of the infection. In the environment of the study, participants felt that disclosure of their status could lead to further disruption of their lives and hence avoided it.

The study has a number of obvious limitations. Only clients who were recently initiated on ART participated in our study and therefore it does not capture the perspectives of ‘treatment-experienced clients’ on the necessity of concealment. It is possible that those who have lived with their HIV-positive status for longer periods become more comfortable over time with it and begin to consider the possibility of disclosure. Disclosure to sexual partners/spouses did not come up as a major issue among the adults who participated in this study, since most of them had already lost their partners. The exception to this, as noted above, was sex workers. Our sample was also limited to women and children and therefore the perspectives of men on ART on concealment and how it affects their lives has not been captured.

Nonetheless, the theme of concealment as a necessity was so common among our interviewees, and has also been informally reported so widely among people living with HIV and health professionals alike in India, that serious consideration should be given to examining ways in which recognition of this perceived requirement may be built into the treatment and care of those living with HIV in this setting.

There is therefore a need to assess the effectiveness of interventions that have been carried out to reduce stigma and discrimination of people living with HIV. Currently, the national treatment programme in India makes provision for peer counselling of diagnosed clients by members of voluntary sector networks of HIV-positive people, some of whom visit ART clinics on a regular basis. However, referral to a peer counsellor is left to the discretion of clinic staff and peer counsellors have no direct access to the clients. Contact with peer counsellors, though considered a good thing, is not seen as an integral part of care activities at the clinic. Mandatory contact with counsellors who are HIV-positive could help those who are newly diagnosed to reduce the felt stigma associated with a diagnosis of HIV. Such counsellors and healthcare workers could also be trained to provide support by helping people who are starting ART to devise strategies to continue to access services and remain on treatment while developing socially acceptable explanations that shield such individuals from stigmatisation.

There is also a pressing need for parents and caregivers of children to receive professional assistance to address the issue of disclosure to children gradually in an age-appropriate manner, in line with WHO (2011) guidelines. This would help them to provide credible answers to children who are keen on understanding what is happening to them and the reasons behind their medication usage, and to better manage and minimise any potentially stigmatising and adverse psychosocial consequences of disclosure for caregivers and children alike.

With the recent attention worldwide given to ‘treatment as prevention’ (Granich et al. 2010; Lancet 2011), there is a growing greater focus on getting more HIV-positive individuals on treatment earlier than had previously been considered clinically advisable. This will in turn require people who are largely asymptomatic to take ART and face either disclosing their status to those in their immediate social networks or coming up with credible alternative explanations for taking medication when they are otherwise healthy. Hence, while designing public health interventions that are meant to promote the health of those living with HIV, as well as to prevent onward transmission, policy makers and programme managers should take into account the social context of people's lives, the consequences of disclosure of HIV status and ways in which negative impacts could be minimised.

Understanding the need of clients to maintain as normal a life as possible, coupled with the association of HIV with deviant sexual behaviour and the stigma attached to it, helps to better explain why many HIV-positive individuals continue to resist efforts made by well-meaning clinical and programme managers to promote disclosure of status and instead go to great lengths to conceal their diagnosis. Discussions of social stigma in relation to HIV have tended to see the reduction of stigma as an end in itself for HIV-positive people, as well as a means of normalising their lives by reducing the likelihood of discrimination (Paxton 2002). While working towards the societal reduction of stigma and elimination of discrimination is indeed essential for social acceptance, our findings demonstrate that at the individual level, strategies based on reducing stigma through disclosure of HIV status fail to engage with the reality that concealment is itself a stigma management strategy.
